# Dental Education from the View of Experience1Read at the Fourth International Dental Congress, St. Louis, August 29 to September 3, 1904.

**Published:** 1905-06

**Authors:** James Truman

**Affiliations:** Philadelphia


### DENTAL EDUCATION FROM THE VIEW OF EXPERIENCE.[1]

BY JAMES TRUMAN, D.D.S., LL.D., PHILADELPHIA.

The following propositions are, in the opinion of the writer, fundamental to a proper realization of ideals in education.

1. A decided mental tendency, in the individual, in one direction.

2. This established, training must be to develop these mental tendencies.

3. Dental education, to be effective, must, therefore, be developed from these original tendencies; if these are in the direction of its complex character, practical and theoretical.

---

[1] Read at the Fourth International Dental Congress, St. Louis, August 29 to September 3, 1904.

4. In this education the *practical* must *precede* the theoretical.

Considering these propositions *seriatim* it must be conceded that the mental tendencies of the individual are primarily the most important for the educator to consider. The study of the mental inclinations of youth belongs not alone to the teacher, but is part of the important relations held by parents and guardians. The latter may regard this a difficult problem to solve, belonging more properly to the specialist in psychological manifestations. To reason thus is a fatal error, and one so often made that it has become a very serious evil, forcing young minds in wrong directions and wrecking innumerable lives in vain attempts to acquire, to them, useless knowledge.

The general idea prevalent—not alone among parents, but as a part of the general educational thought—is that education, to be of value, must run along hard and fast lines, and that without variation, to meet these special tendencies; in other words, the child is placed, as it were, in a straight-jacket from the time of entering school until the period at which the diploma is reached. This may be the A.B. of the high school, or this degree in Arts of our universities or colleges. Is it any wonder that the student whose tastes run counter to all this should regard with aversion this iron-clad method? The average teacher, however, holds that all that is necessary to draw out the best in the individual is to force the undeveloped being through the universal educational mill. It is immaterial to this teacher whether the student is to become a mechanic, an architect, a minister, a lawyer, a doctor, a dentist, or a journalist; in fact, any of the varied and useful occupations that go to make up what we term civilization. To merely name this variety stamps the preliminary work, as usually given, as the weak point in the school training of America. They have a far better idea of education in some parts of Continental Europe, in that the training is in the direction of the future work of the student, but, while this is an advance, it takes but a limited account of the mental tendencies of the individual. It is true that a thorough classical education is of untold value to any individual, and, other things being equal, may give power and marked advantage over his less fortunate competitor in the race of life; but this will depend so much on natural tendencies and the subsequent use made of these, that no one can wisely prophesy the future of the individual.

Two prominent and very recent illustrations in this country serve to give point to this by no means original idea. One, a graduate in arts from one of the leading colleges of the country, found his acquirements of no practical value in the active world. His training, good as it was for certain purposes, furnished nothing that would enable him to reach a position consonant with his tastes and necessities. His learning failed to meet the demands of his daily needs, and, nothing else offering, he bravely accepted the humble position of a laborer on a railway. The result was that step by step he advanced from this to the position of motorman on an electric street-car. From this, the presidency, at an early age, of the system of street railways in the largest city of the country was the natural result of untiring efforts and original mental training in other directions.

The other began with no such education; in fact, his scholastic training was below the average. He started as brakeman on a railroad, and from that, through all grades, became eventually many times a millionaire, a United States Senator, and has recently been nominated for the position of Vice-President of the United States by one of the great political parties of this country. The conclusion to be derived from these examples, not at all uncommon, is that the first demonstrates the power of a liberal education when started in the direction of the natural bent of mind in the individual; and the second, that defects in education may be overcome by mental force concentrated on one object.

If, then, it be true that a mental tendency in the individual is all important and must be sought for, when found it becomes of vital importance that the training should be in the direction of these natural tastes. The problem becomes complicated when the individual exhibits no special proclivities, but seems willing to drift along with the world's current. General laws are not for these, however, and the only test for this very large class of young persons is to prove their capacity in some one or more directions. When, however, the tendency is marked, every effort should be made to begin the individual's career properly. If this is in the direction of mechanics, no mistake will be made if during the impressible period of youth the muscles are placed in training in schools especially devoted to this work. This experience will be of inestimable value should the youth subsequently prefer the literary training necessary to become a doctor of medicine, minister, or lawyer. Especially will this apply to the surgeon.

Dental education, then, to be effective must be based on an early investigation of the mental tendencies, and these being discovered, the direction which education must take is then plain. Dentistry is a complex study. It begins with the practical and ends with the theoretical, or should so end. From what has already been stated, the necessity for early muscular training in mechanics must be apparent. No other course is left open. Every added year lost in this cultivation is just one year of less ability to overcome mechanical problems.

The idea is often taught, and as often combated, that the theoretical must precede the practical; in other words, the individual must have a special training before he is supposed to be fitted to begin his work in dentistry. The Association of Faculties says the applicant for the degree of Doctor of Dental Surgery must first show, on matriculating, a certificate of entrance to the third year of a high school. Nothing is said as to the character of the training given in the high school. Our universities demand a fixed standard, easily understood, and very much higher than the ordinary routine of studies. What is the result? Our dental colleges are inundated with educated young men, who have been sent by those in authority over them to master a calling, a prerequisite to success in which is, at least, a taste for mechanical work. Nothing in their previous studies leads to a knowledge of this bent of mind, and the results, therefore, are not always satisfactory.

To decide this question of fitness for the proposed study, the practical must precede the theoretical, and any other course, as far as dentistry is concerned, is simply a great wrong perpetrated in the name of higher education. No greater mistake can be made than to place a youth in a medical college as preliminary to his dental training. The time may come, indeed, is fast coming, if not already here in some institutions, of carrying the studies of medicine and dentistry *pari-passu.* This will doubtless lead to the M.D. degree, but a much longer period of probation than the present four years of training will be required to secure separately either of the degrees of M.D. or D.D.S. The experience of dental educators has been, and this has been confirmed in the writer's life work, that the individual who has passed from the higher schools into those of medicine, and then essays the study of dentistry, fails, with few exceptions, in reaching a high degree of skill. The evidences of the truth of this are among the most painful of the writer's experiences as a teacher in dentistry, and this alone would lead him to enter an earnest protest against this, to him, fatal error in the future proposed education of dentists.

There has been a general impression that the higher preliminary education, if it did not increase ability in the two practical branches of dentistry, would, at least, enable the student to hold his own and, in other respects, make him more valuable in the community. This view is partially borne out by the averages taken in the Dental Department of the University of Pennsylvania. The following table illustrates the average standing of the several classes in Prosthetics and Operative Dentistry from the period when the Department accepted students upon a grammar-school certificate to the time when a high-school diploma was required.

| Certificate. | Mechanical Dentistry. | Operative Dentistry. |
|---|---|---|
| Grammar school | 76.22 | 84.82 |
| First year high school | 81.19 | 85.91 |
| Second year high school | 83.13 | 80.97 |
| Diploma, high school | 75.47 | 86.54 |

While averages are somewhat misleading, yet, so far as this goes, as the result in one school, it may be taken as an indication that the boy from the grammar schools stands an equal chance with the boy with a high school diploma in mastering the practical work of dentistry, but it is very evident that the higher education has not improved the high school boy's ability in meeting the difficulties of the practical over the one of the lower. It, however, does show that the higher preliminary training enables the one who has it to hold his own in the realms of the practical and will give him an immense advantage in acquiring the collateral branches peculiar to medicine.

In conclusion, dental education must be first based on mental tendencies, and, these proved, then the education should begin with manual training work. It is immaterial where this is secured. From this the practical side of dentistry, the prosthetic, must be conquered. This will furnish the foundation for the operative branch, and these two constitute the necessary practical for the attainment of success in the future professional life work of the individual. It must, however, never be forgotten that the old age of manual dexterity begins with maturity, and skill, to be of value and permanent, must be acquired while the mind and the hands are in the impressive period of youth.

